# A cluster of metabolism-related genes predict prognosis and progression of clear cell renal cell carcinoma

**DOI:** 10.1038/s41598-020-67760-6

**Published:** 2020-07-31

**Authors:** Mei Liu, Qiufeng Pan, Ruihai Xiao, Yi Yu, Wenbao Lu, Longwang Wang

**Affiliations:** 1 Department of Anesthesiology, The First Affiliated Hospital of Nanchang University, Nanchang, China; 2 Department of Urology, The First Affiliated Hospital of Nanchang University, Nanchang, China; 3 Department of Urology, Affiliated Hospital of Jiangxi Academy of Medical Sciences of Nanchang University, Nanchang, China; 4 Department of Urology, The Second Affiliated Hospital of Nanchang University, Nanchang, China; 5 Department of Urology, Jiujiang University Affiliated Hospital, Jiujiang, China

**Keywords:** Cancer metabolism, Gene regulation, Cancer, Cell biology, Genetics, Biomarkers

## Abstract

Clear cell renal cell carcinoma (ccRCC) has long been considered as a metabolic disease characterized by metabolic reprogramming due to the abnormal accumulation of lipid droplets in the cytoplasm. However, the prognostic value of metabolism-related genes in ccRCC remains unclear. In our study, we investigated the associations between metabolism-related gene profile and prognosis of ccRCC patients in the Cancer Genome Atlas (TCGA) database. Importantly, we first constructed a metabolism-related prognostic model based on ten genes (ALDH6A1, FBP1, HAO2, TYMP, PSAT1, IL4I1, P4HA3, HK3, CPT1B, and CYP26A1) using Lasso cox regression analysis. The Kaplan–Meier analysis revealed that our model efficiently predicts prognosis in TCGA_KIRC Cohort and the clinical proteomic tumor analysis consortium (CPTAC_ccRCC) Cohort. Using time-dependent ROC analysis, we showed the model has optimal performance in predicting long-term survival. Besides, the multivariate Cox regression analysis demonstrated our model is an independent prognostic factor. The risk score calculated for each patient was significantly associated with various clinicopathological parameters. Notably, the gene set enrichment analysis indicated that fatty acid metabolism was enriched considerably in low-risk patients. In contrast, the high-risk patients were more associated with non-metabolic pathways. In summary, our study provides novel insight into metabolism-related genes’ roles in ccRCC.

## Introduction

Clear cell renal cell carcinoma (ccRCC), the most common renal cell carcinoma (RCC) subtype, exhibits global health issues due to its growing incidence, extreme heterogeneity among patients and high mortality. It is estimated that 90% of the ccRCC patients died of tumor-specific recurrence and metastasis^1^. Regarding this, considerable research efforts have focused on developing a model to predict the prognosis of ccRCC patients; however, these prognosis tools still require improvements to attain a high degree of accuracy^2–4^. Therefore, novel and robust prognostic models are urgently needed in clinical practice.

Metabolism is fundamental in maintaining all the biological processes necessary for life^5^. Tumors are typified by metabolic abnormalities as proliferating cells rewire their mechanism to sustain growth^6^. Notably, the rapidly proliferating cells in malignancies take up abundant glucose and glutamine to generate the proteins, lipids, and nucleic acids to support cell growth^7^. RCC is regarded as a metabolic disease with diabetes, obesity, and atherosclerosis considered as the risk factors^8,9^. CcRCC is a unique RCC subtypes based on the abnormally accumulated lipid droplets in the cytoplasm with research evidence implicating the lipid accumulation in disease progression^10,11^. Nevertheless, the underlying mechanism and the prognostic role of these metabolic genes remain largely unknown.

In the present study, we screened the differentially expressed metabolism-related genes and evaluated their clinical value based on the cancer genome atlas (TCGA) kidney renal clear cell carcinoma (KIRC) cohort. Subsequently, we selected a cluster of metabolism-related genes to construct a prognosis prediction signature using the least absolute shrinkage and selection operator (LASSO) regression algorithm. This signature was further validated in the clinical proteomic tumor analysis consortium (CPTAC) database. Moreover, our prognosis prediction could serve as an independent prognostic factor. Importantly, we investigated the underlying mechanism using the gene set enrichment analysis (GSEA) database and the Metascape database^12^.

## Results

### Construction of a risk signature associated with metabolism

**Figure 1:**
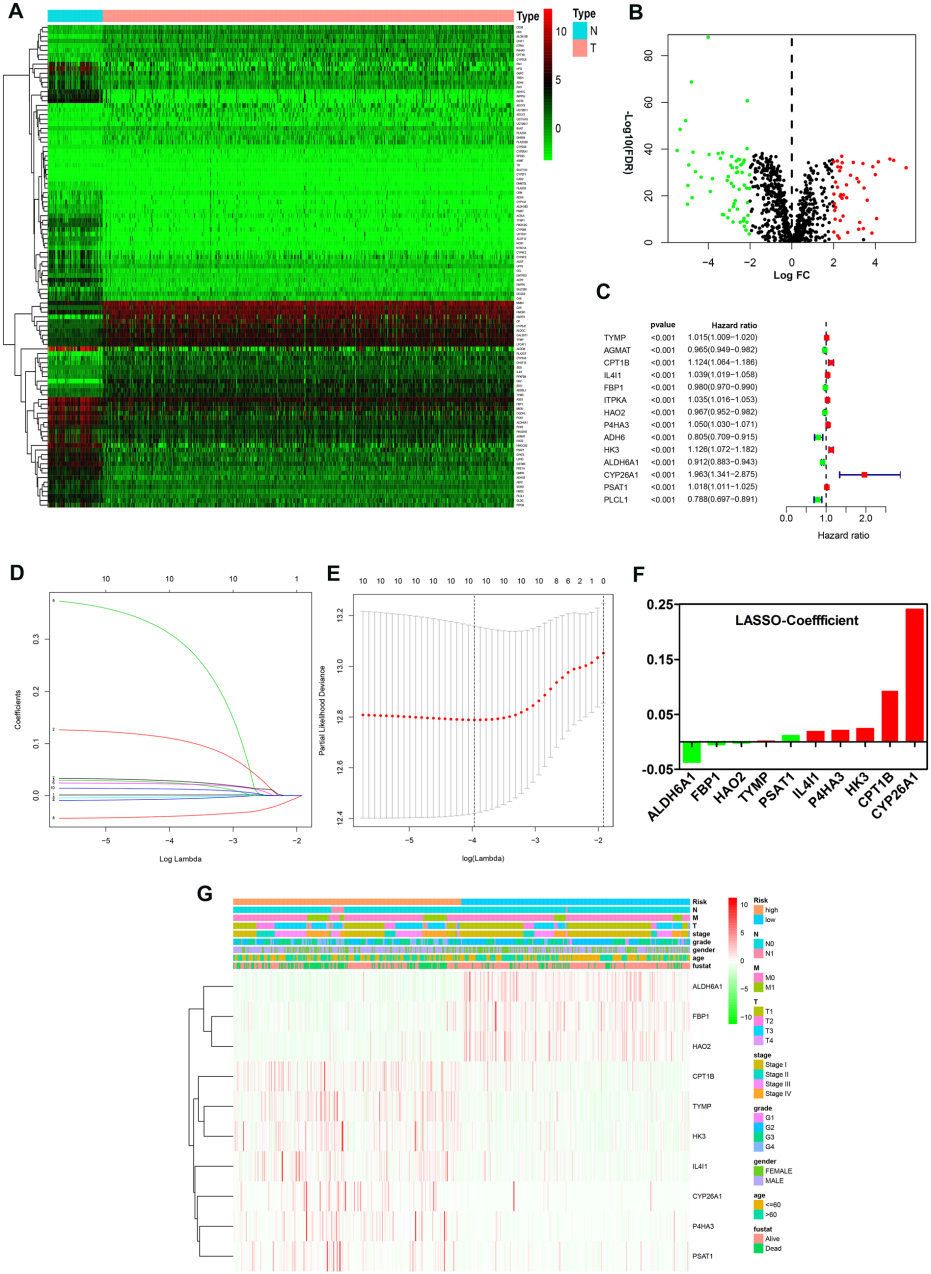
Identification of a metabolism-related signature by Cox proportional hazards model in TCGA cohort. (**A**,**B**) Heatmap and Volcano plot of 105 differentially expressed metabolism-related genes. (**C**) Forrest plot of results of the univariate Cox regression analysis (*P* < 0.001). (**D**,**E**) Lasso Cox regression algorithm. (**F**) Coefficient value for each of the 10 active covariates genes (red represents upregulated in tumor, while green indicates the downregulated), X-axis represents gene name, and Y-axis represents the coefficient value of the model. (**G**) Heatmap of the 10 genes of the signature based on the risk score value.

**Figure 2:**
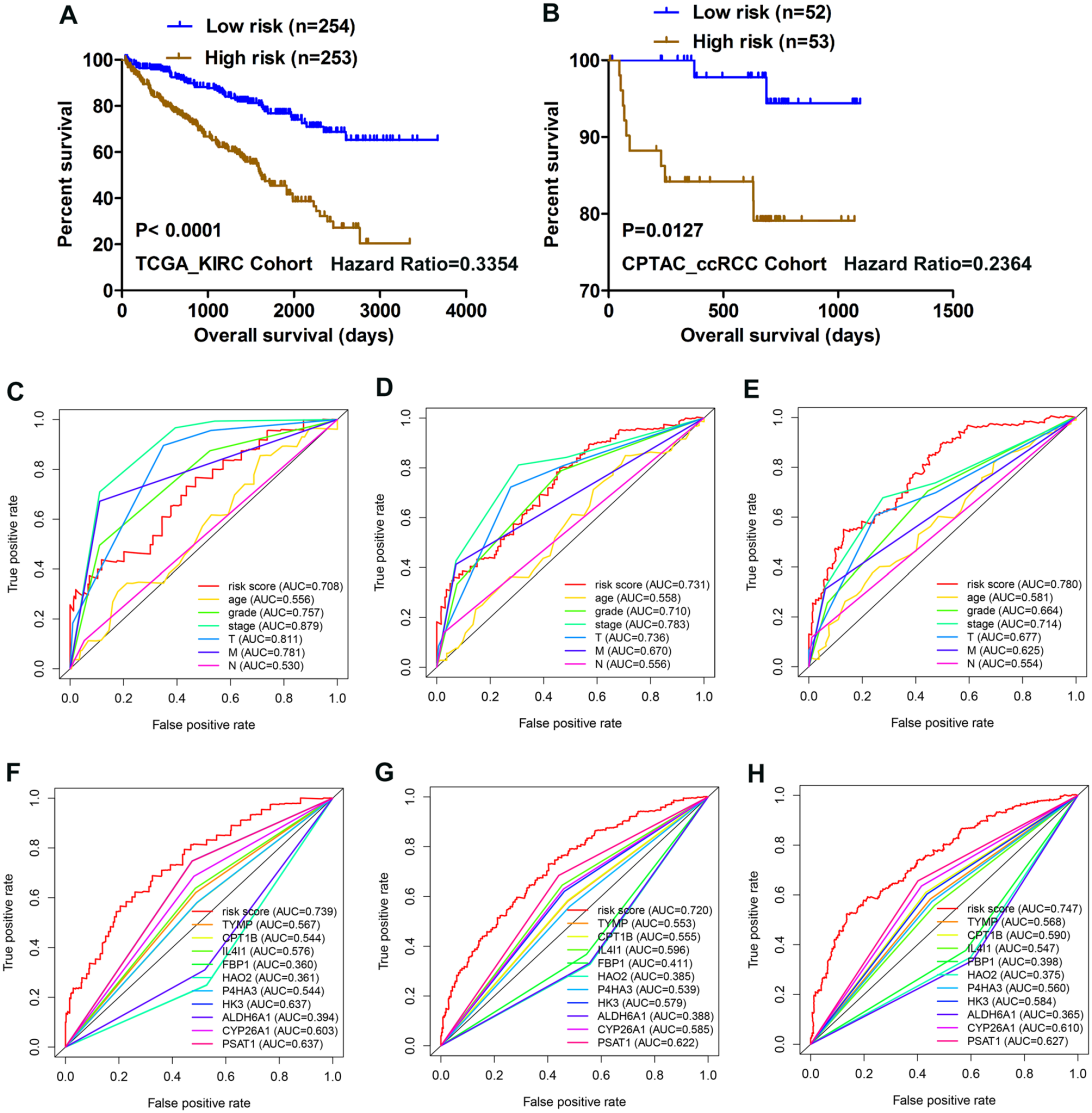
Kaplan–Meier analysis and time-dependent ROC analysis for the 10-gene signature in ccRCC. (**A**) Kaplan–Meier curve with Log-rank test in TCGA_KIRC Cohort. (**B**) Kaplan–Meier curve with Log-rank test in CPTAC_ccRCC Cohort. (**C**–**E**) ROC curve of model and clinical characteristics predicting 1-, 3- and 5-year survival based on TCGA training set. (**F**–**H**) ROC curve of model and single gene predicting 1-, 3- and 5-year survival based on TCGA training set.

A total of 105 differentially expressed metabolism-related genes were found between normal samples and tumor samples in TCGA_KIRC cohort (adj. *P* < 0.05 and |logFC|> 2). Specifically, 61 genes were downregulated, while 44 genes were upregulated (supplementary Table 1); the corresponding heatmap plot and volcano plot are shown in Fig. 1A,B. After that, we identified 12 genes (Fig. 1C) that were significantly associated with the patient’s overall survival (*P* < 0.001) by univariate Cox regression analysis; Lasso Cox regression algorithm was then utilized to screen out 10 genes as active covariates in evaluating the risk score for the patients (Fig. 1D–F). The ten genes included in the model were Fructose-Bisphosphatase 1 (FBP1), Hydroxyacid Oxidase 2 (HAO2), Phosphoserine Aminotransferase 1 (PSAT1), Aldehyde Dehydrogenase 6 Family Member A1 (ALDH6A1), Thymidine Phosphorylase (TYMP), Interleukin 4 Induced 1 (IL4I1), Prolyl 4-Hydroxylase Subunit Alpha 3 (P4HA3), Hexokinase 3 (HK3), and Carnitine Palmitoyltransferase 1B (CPT1B), Cytochrome P450 Family 26 Subfamily A Member 1 (CYP26A1). The Kaplan–Meier survivor analysis of the ten genes was plotted based on an online database called “ONCOLNC” (https://www.oncolnc.org) (supplementary figure 1). The risk score for each patient was calculated as follows: − 0.03677*Expression of ALDH6A1-0.00467* Expression of FBP1-0.001642* Expression of HAO2 + 0.001219* Expression of TYMP + 0.01105* Expression of PSAT1 + 0.018708* Expression of IL4I1 + 0.020459* Expression of P4HA3 + 0.023879*Expression of HK3 + 0.092914* Expression of CPT1B + 0.240626* Expression of CYP26A1. We separated patients into high- and low-risk groups based on the median risk score. Heatmap plot was depicted to reveal the clinicopathological characteristics between the low-risk and high-risk patients (Fig. 1G). The overall survival (OS) of the high-risk group was significantly poorer compared with the low-risk group in TCGA_KIRC Cohort (*P* < 0.0001; Fig. 2A). Moreover, this model was further validated in the CPTAC_ccRCC Cohort; patients in the low-risk group had a better prognosis than in the high-risk group (*P* < 0.05; Fig. 2B). The clinical characteristics of CPTAC_ccRCC Cohort patients are in supplementary table 2. The area under the curve (AUC) for 1-, 3-, and 5-year OS of risk score compared with age, grade, TNM stage, T, M and N were 0.708, 0.731, and 0.780 respectively (Fig. 2C–E, supplementary figure 2); the AUC for 1-, 3-, and 5-year OS of risk score compared with ALDH6A1, FBP1, HAO2, TYMP, PSAT1, IL4I1, P4HA3, HK3, CPT1B, and CYP26A1 were 0.739, 0.720, and 0.747 respectively (Fig. 2F–H). Altogether, these results indicated that the ten genes form a potential robust prognostic model. Finally, we constructed a predictive nomogram by combing the TNM stage, age, grade, T, M, N and the prognostic model (supplementary figure 3).

### Independence role of the prognostic model

**Figure 3:**
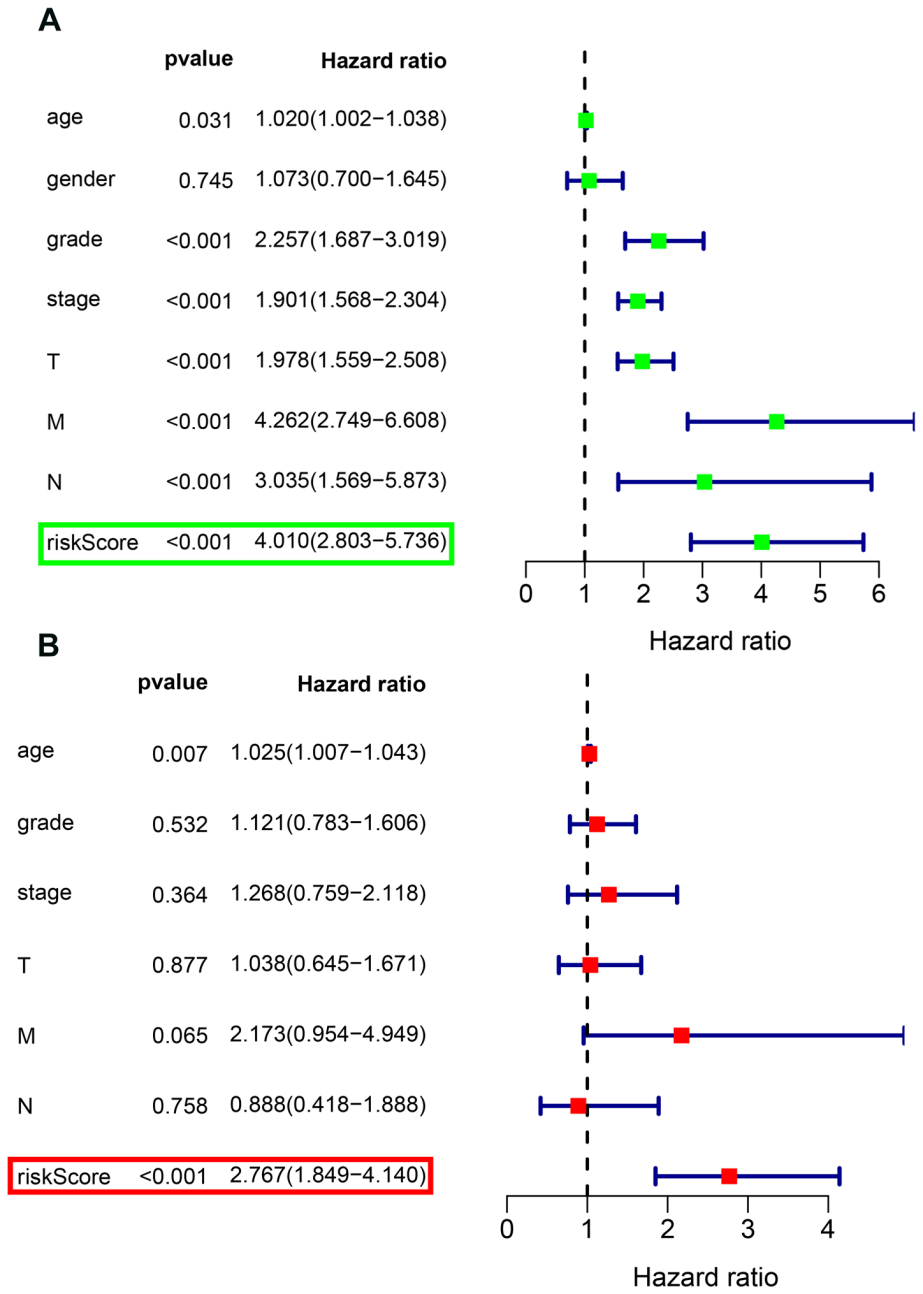
Forrest plot of the univariate and multivariate Cox regression analysis in TCGA_KIRC Cohort. (**A**) The univariate Cox proportional regression analysis. (**B**) The age, grade, stage, T stage, N stage, M stage and risk score were adjusted in our multivariate Cox regression model.

First, univariate Cox regression analysis indicated that age, grade, stage, T, M, N and risk score were correlated with OS (Fig. 3A). Subsequently, multivariate Cox regression analysis adjusted for age, grade, stage, T, M, N and risk score revealed that risk score could serve as an independent prognostic factor (*P* < 0.001, Hazard Ratio:1.849–4.14) (Fig. 3B).

### Patients with a higher risk score had a poorer clinical outcome

**Figure 4:**
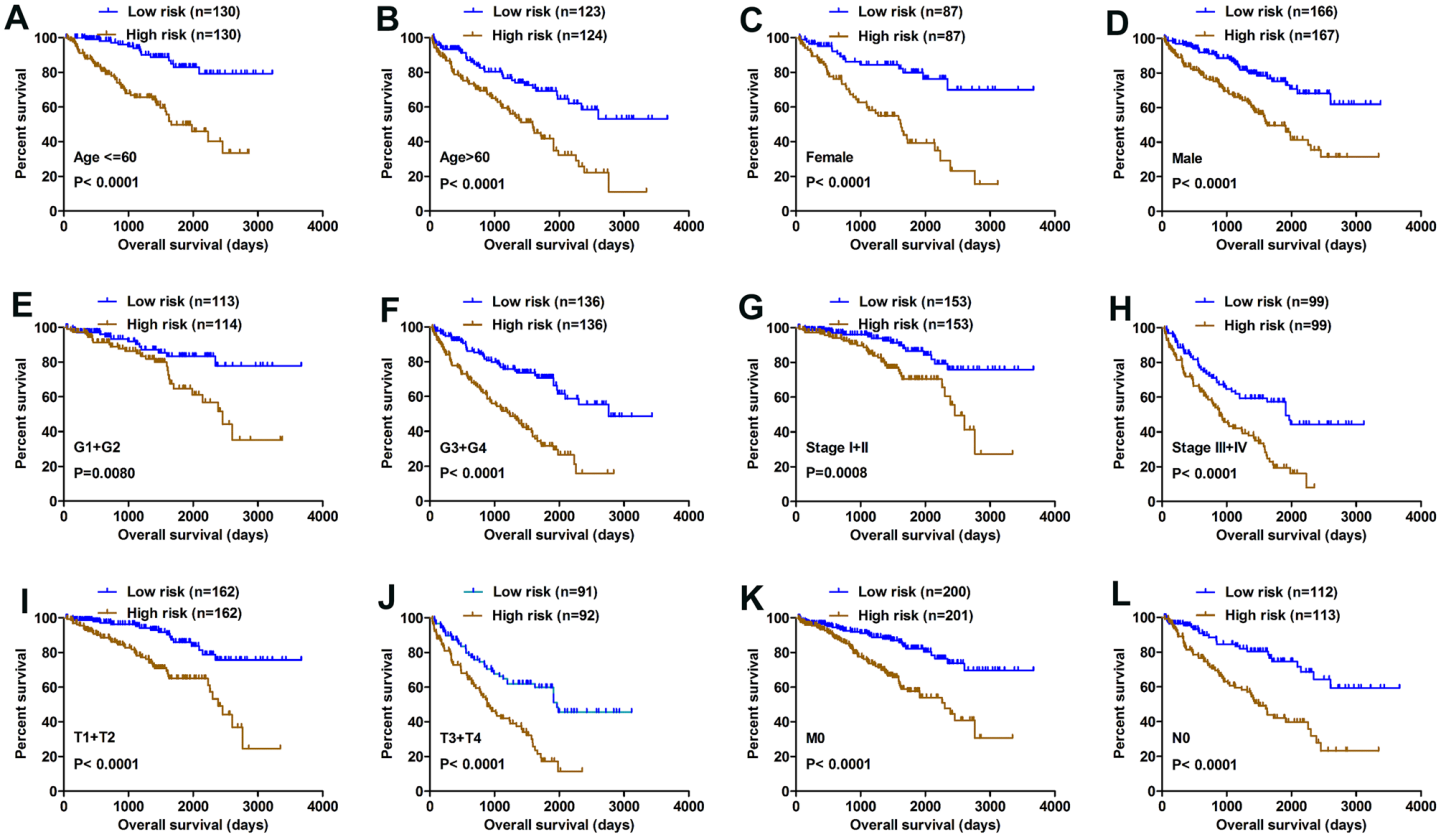
Kaplan–Meier analysis of subgroup patients based on risk scores. (**A**–**L**). The Kaplan–Meier curve revealed the survival of patients was significantly poorer in subgroup patients with high risk scores.

The Kaplan–Meier survival analysis was applied to determine OS in different subgroups of patients according to the risk score level. The results demonstrated that risk score may be a potential prognostic biomarker for patients with the following characteristics: aged ≤ 60 or > 60 years old (Fig. 4A,B), Female or Male (Fig. 4C,D), G1 + G2 or G3 + G4 (Fig. 4E,F), Stage I + II or Stage III + IV (Fig. 4G,H), T1 + T2 or T3 + T4 (Fig. 4I,J), M0 (Fig. 4K) and N0 (Fig. 4L).

### The risk score system was associated with various clinicopathological parameters

**Figure 5:**
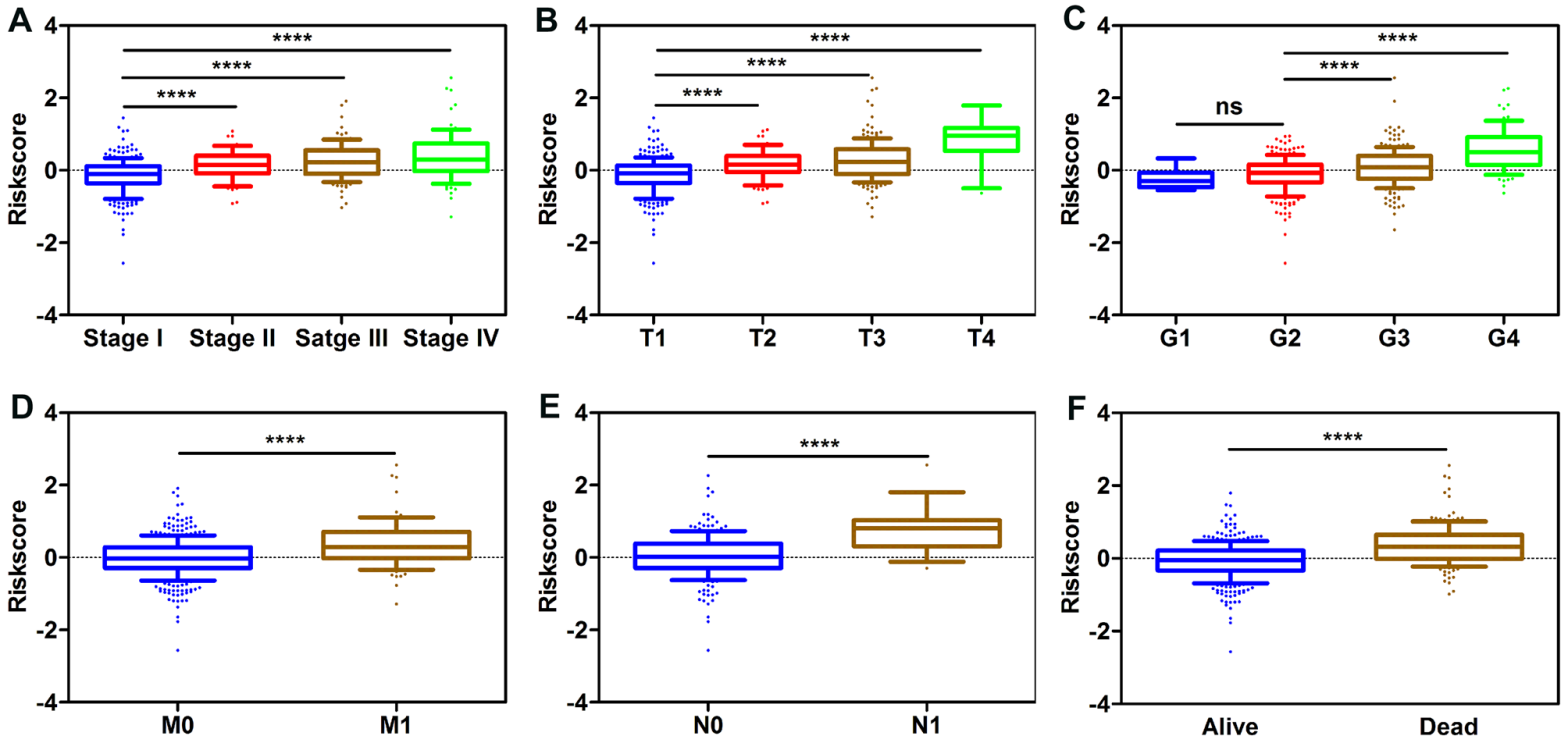
Risk score was closely associated with various clinicopathological characteristics. (**A**) Stage. (**B**) T stage. (**C**) Grade. (**D**) M stage. (**E**) N stage. (**F**) Status.

We explored the correlation between the risk score and different clinicopathological factors. Chi-square test revealed that the risk scores were significantly associated with patients’ gender, histological grade, TNM stage, T stage, N stage, M stage and Vital status (Table 1). Furthermore, subgroup analysis confirmed that higher risk scores were significantly correlated with higher TNM stage, higher tumor T stage, distant metastasis, lymph node metastasis, and higher histologic grade (Fig. 5A–F).

**Table 1:** Associations between risk model and clinicopathological parameters of patients.

Parameters	TCGA_KIRC cohort		χ^2^	*P*-value
	Low risk (n = 254)	High risk (n = 253)		
**Age** **(years)**				
≤ 60	133	127	0.238	0.626
> 60	121	126		
**Gender**				
Female	150	183	9.912	0.002
Male	104	70		
**Grade**				
G1 + G2	147	79	36.433	< 0.001
G3 + G4	107	174		
**T stage**				
T1 + T2	193	131	32.196	< 0.001
T3 + T4	61	122		
**N stage**				
N0 + Nx	252	239	9.342	0.002
N1	2	14		
**M stage**				
M0 + Mx	231	198	15.665	< 0.001
M1	23	55		
**Stage**				
I + II	188	118	39.698	< 0.001
III + IV	66	135		
**Status**				
Dead	207	138	42.341	< 0.001
Alive	47	115

### RT-qPCR and external validation of expression level

**Figure 6:**
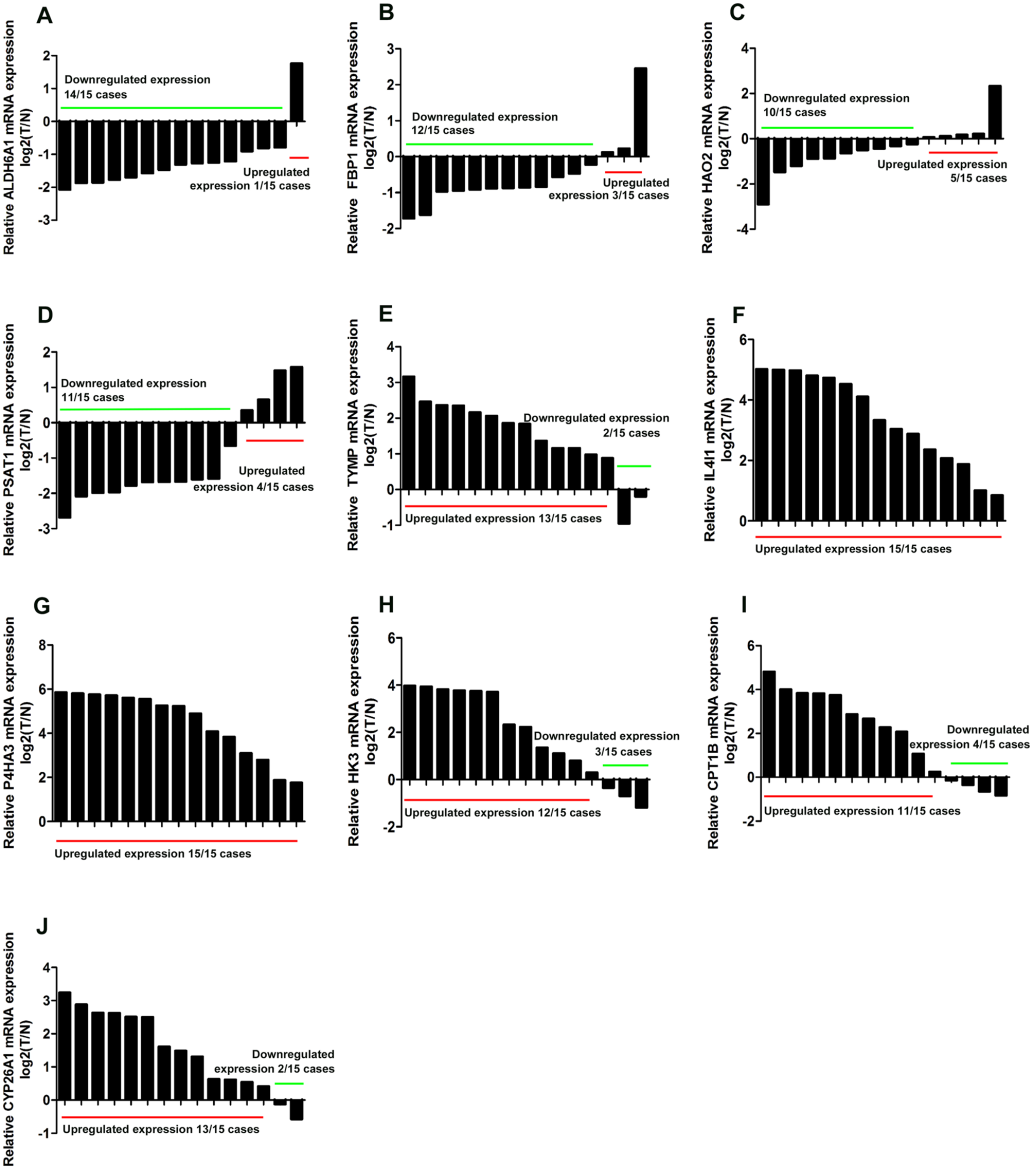
RT-qPCR verification of the expression level of 10 genes. (**A**) ALDH6A1, 14 cases with downregulated expression compared with corresponding normal tissue. (**B**) FBP1, 12 cases with downregulated expression compared with corresponding normal tissue. (**C**) HAO2, 10 cases with downregulated expression compared with corresponding normal tissue. (**D**) PSAT1, 11 cases with downregulated expression compared with corresponding normal tissue. (**E**) TYMP, 13 cases with upregulated expression compared with corresponding normal tissue. (**F**) IL4I1, 15 cases with upregulated expression compared with corresponding normal tissue. (**G**) P4HA3, 15 cases with upregulated expression compared with corresponding normal tissue. (**H**) HK3, 12 cases with upregulated expression compared with corresponding normal tissue. (**I**) CPT1B, 11 cases with upregulated expression compared with corresponding normal tissue. (**J**) CYP26A1, 13 cases with upregulated expression compared with corresponding normal tissue. Log_2_T/N: − △△CT.

**Figure 7:**
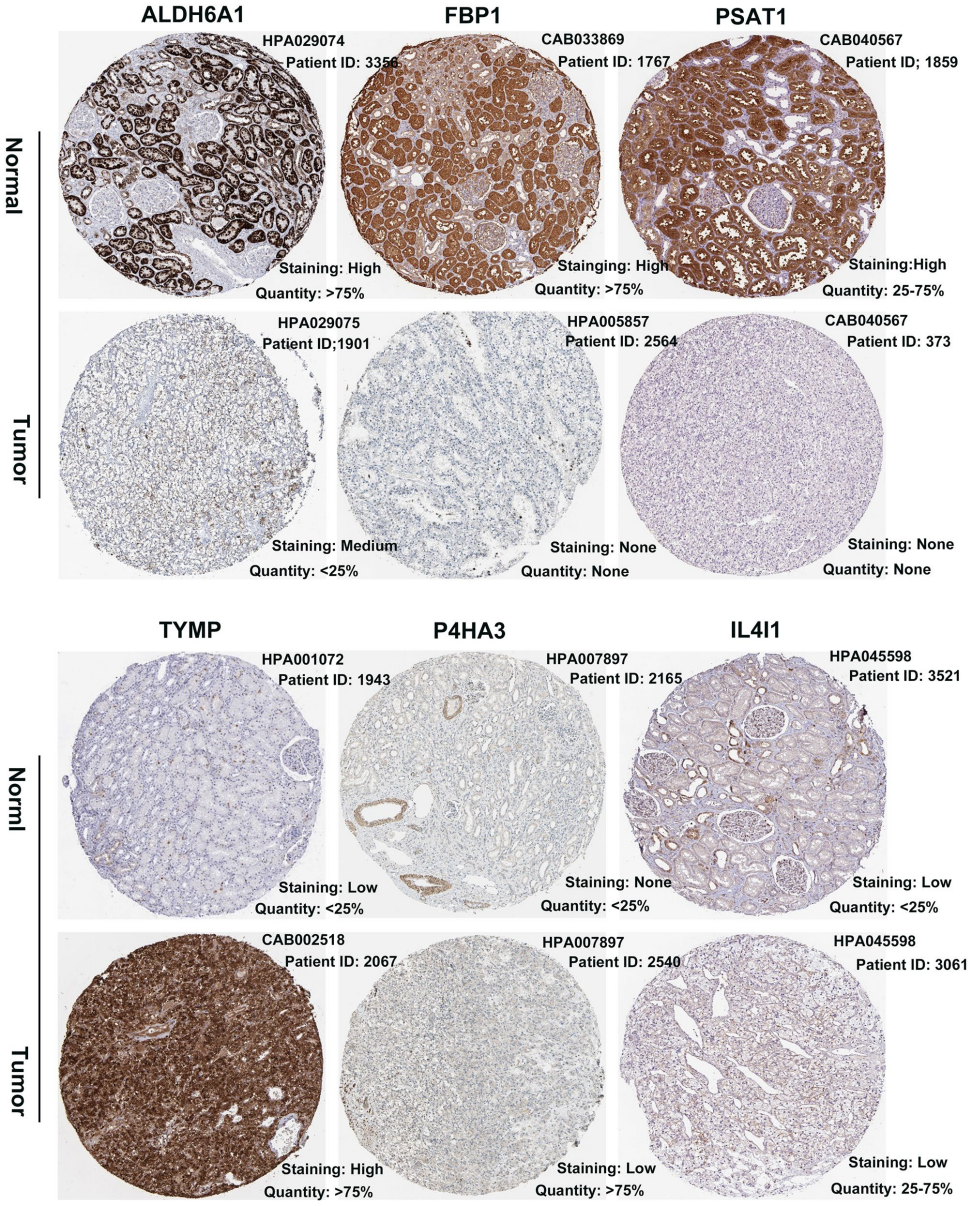
The protein expression of six predictive genes. The representative protein expression of the six gene in ccRCC and normal kidney tissue, four genes were not found. Data was retrieved from the Human Protein Atlas (https://www.proteinatlas.org). ALDH6A1, FBP1, and PSAT1 were significantly underexpressed in ccRCC. While TYMP, P4HA3 and IL4I1 were found significantly overexpressed in ccRCC.

We used online database to validate the expression level of the 10 genes constructing our prognostic model. Consistent with our results, ALDH6A1, FBP1, HAO2 and PSAT1 were found to be significantly downregulated in tumor samples compared with normal samples in both Oncomine and TIMER database (supplementary figure 3A,B); TYMP, HK3, and P4HA3 were found to be significantly overexpressed in tumor samples in both Oncomine and TIMER database, while IL4I1 and CYP26A1 were found over-expressed only in TIMER database (supplementary figure 4A,B). Meanwhile, RT-qPCR assays revealed that ALDH6A1, FBP1, HAO2 and PSAT1 were markedly under-expressed in tumor tissues in exceeding 60% cases, and that TYMP, HK3, P4HA3, IL4I1, CYP26A1 and CPT1B were over-expressed in tumor tissues in exceeding 70% cases (Fig. 6A–J). Furthermore, we retrieved the HPA database and found that the protein expression of ALDH6A1, FBP1 and PSAT1 were downregulated in KIRC; the protein expression level of TYMP, P4HA3 and IL4I1 were upregulated in KIRC (Fig. 7).

### Functional annotation of 10-gene signature

**Figure 8:**
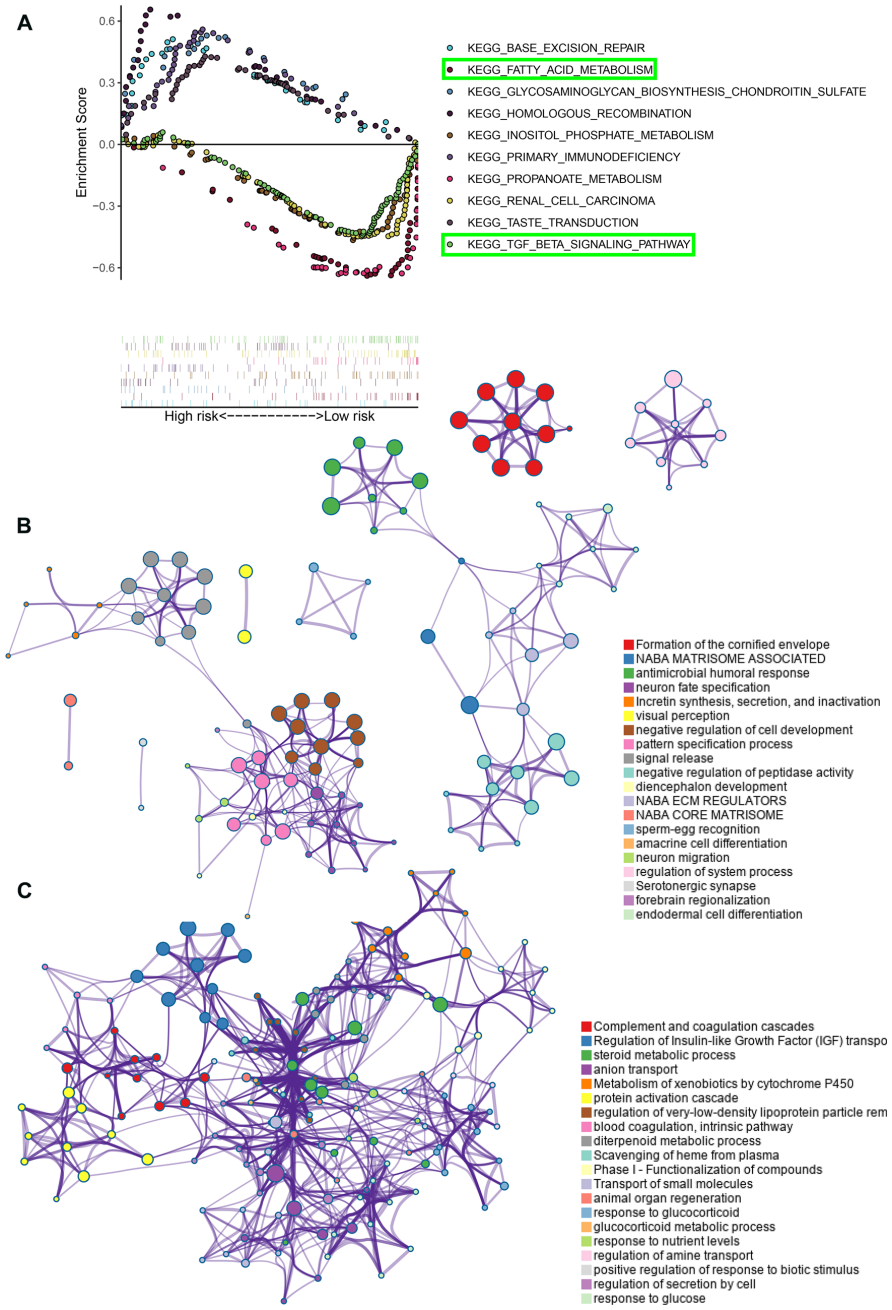
The functional annotation of the 10-gene signature. (**A**) Ten representative KEGG pathway in high-risk and low-risk patients. (**B**) Functional annotation of the upregulated genes in high-risk patients with Metascape database. (**C**) Functional annotation of the downregulated genes in high-risk patients with Metascape database. Balls together with the same color indicates one biological process.

To investigate the potentially underlying mechanism between the two risk groups, GSEA was performed in TCGA_KIRC cohort and we found 34 significantly enriched pathway. Top 10 enriched KEGG pathway were shown in Fig. 8A. Fatty-acid metabolism, TGF-βsignaling pathway, Renal cell carcinoma, Propanoate metabolism and Inositol-phosphate metabolism were significantly enriched in low risk group patients. Moreover, differentially expressed genes analysis between low risk patients and high risk patients revealed that 96 were downregulated and 400 were upregulated in high risk patients (adj. *P*-value < 0.05 and |logFC|> 2); “Metascape” analysis indicated that top 20 enriched clusters of downregulated genes and upregulated genes (Fig. 8B,C; supplementary figure 5).

## Discussion

Globally, RCC accounts for more than 2% of neoplasms in humans worldwide with the incidence and mortality persistently increasing^1^. Recently, gene signatures based on specific characteristic-related to predict prognosis have become a hotspot in cancer research^13–15^. In the present study, to the best of our knowledge, we constructed the first novel metabolism-related risk signature with the potential application of predicting the prognosis and progression of ccRCC.

We identified a novel robust 10-gene metabolic prognostic signature based on the TCGA_KIRC dataset and further validated it in CPTAC_ccRCC dataset. Our risk score model could efficiently stratify patients’ survival with various clinicopathological parameters and be an independent prognostic factor; moreover, the risk scores increased with the patients’ increasing malignancy. Besides, the performance of our model in the prediction of the 5-year overall survival was superior to other parameters, indicating that it might facilitate the development of a long-term follow-up plan; meantime, the prediction ability of 1-, 3- and 5-years overall survival surpassed a single gene. Altogether, these results revealed our 10-gene metabolic prognostic signature has a great potential in ccRCC. However, in the future, validating our signature in more independent cohorts is still required.

We performed GSEA analysis to investigate the underlying molecular mechanism of the signature. Notably, we found out that fatty acid metabolism was significantly enriched in the low-risk patients, while the high-risk patients were more associated with the non-metabolic pathways. Research evidence reveals that abnormal accumulation of lipid droplets plays a crucial role in ccRCC progression^16^. Our study provided insights by suggesting that low-risk patients could benefit more from fatty acid metabolism targeted therapy in the future. Moreover, the gene annotation and analysis results revealed that overexpressed genes in the high-risk patients are involved in incretin synthesis, secretion, and inactivation, and that the down-regulated genes participate in complement and coagulation cascades, metabolism of xenobiotics by cytochrome P450, response to glucose. Hua et al*.*^17^ identified an immune-related risk signature for predicting prognosis of ccRCC, they found that tumors from high-risk patients had higher relative abundance of T follicular helper cells, regulatory T cells than low-risk patients. Moreover, the time-dependent ROC analysis revealed that the AUC was 0.753, 0.686, and 0.637 at 1, 3, and 5 years. However, in our study, we extracted a cluster of metabolism-related genes and constructed a multi-gene signature, the AUC at 3 and 5 years were 0.731 and 0.780 respectively, which exhibit a better predictive performance.

Most of the genes in our signature have previously been shown to be involved in cancers. However, several genes’ role in ccRCC remain unclear. The Fructose-Bisphosphatase 1 (FBP1) is a rate-limiting enzyme in gluconeogenesis, which is suppressed in kidney tumors and thus feeds ccRCC; FBP1 is associated with impaired cell proliferation, glycolysis and the pentose phosphate pathway in ccRCC in a catalytic-activity-independent manner via direct interaction with the HIF inhibitory domain^18^. PSAT1 is up-regulated in many cancers and acts as an oncogene that exerts a vital role in cancer progression and metastasis^19–21^. Interestingly, through mining public database and RT-qPCR, we found that PSAT1 in ccRCC patients is down-regulated compared with normal tissue. In the future, we will further investigate the underlying mechanism. Hydroxyacid oxidase 2 (HAO2) encodes peroxisomal proteins with 2-hydroxy acid oxidase activity, which participated in the production of reactive oxygen species and cellular breakdown^22^; HAO2 has already been shown downregulated in hepatocellular carcinoma (HCC) tissues and ccRCC, overexpression of which restrained HCC and RCC cells proliferation by eliminating lipid droplet accumulation^23,24^. ALDH6A1 was found significantly reduced in metastatic prostate cancer according to the immunochemistry and western blot results^25^. Our results also revealed that ALDH6A1 is suppressed in ccRCC, which predicts poor prognosis. TYMP is a rate-limiting enzyme in the thymine catabolic pathway and contributes to tumor angiogenesis^26,27^. Prolyl 4-Hydroxylase Subunit Alpha 3 (P4HA3) is a critical enzyme in maintaining the stability of newly synthesized collagen. Song H et al. indicated that P4HA3 is upregulated in gastric cancer and epigenetically activated by slug^28^. In the present study, we demonstrated that P4HA3 is upregulated in ccRCC, and that its high expression reflected poor outcome. IL4I1, an immune-associated gene, inhibited CD8 + T-cells and thus exerted immunosuppressive functions^29^. Our results first revealed that IL4I1 was upregulated in ccRCC, and that low expression of IL4I1 indicated favorable prognosis. HK3 encodes hexokinase 3, upregulation of which is associated with epithelial-mesenchymal transition and may be a metabolic adaptation for colorectal cancer progression^30^. However, the HK3′s role in ccRCC remains unknown. CPT1B is the rate-controlling enzyme in the long-chain fatty acid beta-oxidation pathway in mitochondria. In bladder cancer, Vantaku et al.^31^ demonstrated the suppression of CPT1B inhibits cell proliferation, metastasis in vivo. However, to the best of our knowledge, the role of CPT1B in ccRCC has not been understood. In our study, we showed that CPT1B was significantly upregulated in ccRCC, which was associated with poor prognosis. CYP26A1 (cytochrome P450, family 26, subfamily A, polypeptide 1), a retinoic acid-metabolizing enzyme, is a crucial regulator of cell proliferation, differentiation and apoptosis that is overexpressed in many types of tumors^32,33^. However, one major limitation of our study is that our research regarding the ten genes constructing our signature is insufficient, further intensive studies are required; moreover, despite the robust performance of our model in predicting survival, future validation in clinical practice is needed.

In summary, for the first time, we identified a 10-gene risk signature related to metabolism to independently predict the prognosis of patients with ccRCC; this signature may provided guidance for targeted therapy and potential biomarkers in the future.

## Materials and methods

### Data acquisition

The messenger RNA (mRNA) sequencing data and corresponding clinical data of ccRCC patients were obtained from TCGA_KIRC Cohort of TCGA database. Similarly, the CPTAC protein expression profile of ccRCC and clinical data were downloaded from CPTAC database as validation dataset. The detailed characteristics of patients were presented in Table 1. Moreover, metabolism-related gene set was extracted from the Molecular Signature Database v5.1 (MSigDB) (https://www.broad.mit.edu/gsea/msigdb). Only those genes that were common expressed in these three database were selected for further prognostic analysis.

### The prognostic metabolic gene signature construction

930 annotated metabolic protein-coding genes were utilized for the differentially expressed analysis with the “Limma” version-3.6.1 R package^34^. Then, we performed univariate Cox regression analysis and Lasso-cox regression analysis to identify the prognostic-related metabolic genes and construct a metabolic-related gene signature^35^. A *P*-value < 0.001 in univariate Cox regression analysis was treated as statistically significant. We then computed the risk score for each patients as follows: Risk score = (expression of mRNA1 *Coefficient _mRNA1_) + (expression of mRNA2 *Coefficient _mRNA2_) + (expression of mRNAn *Coefficient _mRNAn_)^36^. According to the median risk value, patients in both TCGA_KIRC Cohort and CPTAC_ccRCC Cohort were separated into low- and high-risk group; The Log-rank test and Kaplan–Meier survival curve were used to assess the prognostic value. Furthermore, we investigated the time-dependent prognostic significance of the gene signature by the R package “survivalROC” ^37^. Meanwhile the independence of the prognostic gene signature in TCGA_KIRC cohort was evaluated by multivariate analysis; the associations between risk score and various clinicopathological parameters were also assessed. A predictive nomogram was built including clinicopathological characteristics and risk score by the R package “rms” ^38^. A detailed flow chart was shown in supplementary figure 6.

### Bioinformatics analysis

To further validate the expression of the prognostic genes constructing the gene signature, we retrieved the Oncomine database (https://www.oncomine.org/), Timer database (https://cistrome.shiyapps.io/timer/) and the human protein atlas (HPA) database (https://www.proteinatlas.org/). To explore the possible underlying mechanism, we performed differentially expressed gene analysis between low risk patients and high risk patients with “edgeR” package^39^; GSEA analysis were conducted to reveal enriched terms; upregulated genes and downregulated genes were put into the gene annotation and analysis resource “Metascape database” (https://metascape.org/gp/index.html#/main/step1) to obtain functional annotation of these genes.

### RNA extraction and RT-qPCR

15 paired kidney cancer and adjacent normal tissues were collected between 2016 and 2018 at the Department of Urology, The First affiliated Hospital of Nanchang University (Nanchang, China). The patients whose tissues were used in the present study had never received chemotherapy or radiotherapy. The specimens were stored at − 80 °C until use. The study protocol was approved by the ethics committee of The First Affiliated Hospital of Nanchang University, and all patients provided written informed consent. The study methodologies conformed to the standards set by the Declaration of Helsinki. Total RNA was isolated from 15 paired tissues using TRIzol reagent (Thermo Fisher Scientific, Inc.)^40^. Then, we detected the concentration and purity of the RNA solution using a NanoDrop 2000 spectrophotometer (NanoDrop Technologies; Thermo Fisher Scientific, Inc.). Before the RT-qPCR, extracted RNA was reverse transcribed into cDNA utilizing Superscript II reverse transcription kit (Takara Bio, Inc.). The reaction conditions were set as follows: 37 °C for 15 min; 85 °C for 5 s. Finally, the cDNA was subjected to reverse transcription PCR using a SYBR-Green master kit (ChamQ; Vazyme, Nanjing, China) on a LightCycler 480 II (Roche Diagnostics); The PCR conditions were as follows: Pre-denaturation at 95 °C for 3 min; 40 cycles of denaturation at 95 °C for 3 s; annealing and extension at 60 °C for 20 s. The housekeeping gene, GAPDH, was used to normalize the relative expression of genes as an endogenous control by the comparative Cq (threshold cycle) method (2^−∆∆Cq^). All RT-qPCR reactions were performed in duplicate. The primers used to amplify and GAPDH were chemically synthesized by TSINGKE. The primer sequences were as follows: TYMP: 5′-GCTCTGGCTCAGCGGAC-3′ (forward) and 5′-GGCGTTCTGCGGGACTTC-3′ (reverse); FBP1: 5′-CACAGCAGTACCCTGACCTG-3′ (forward) and 5′-TTGGTTAGGGAGTGCCAAGC-3′ (reverse); PSAT1: 5′-CCGCGTATTTTGCCTTTGCT-3′ (forward) and 5′-CCTACCCTCTGTGCTGTGTG-3′ (reverse); ALDH6A1: 5′-CTCACCTGCTGTTGTCATGC-3′ (forward) and 5′-GCGGGGTTAATAGTCCTGA-3′ (reverse); and P4HA3: 5′-GCAGCCCCCTCTACAGAATG-3′ (forward) and 5′-CACGCTGAGGTTGGCATAGA-3′ (reverse); and IL4I1: 5′-AGGGTGGCTTTGTGGTACAG-3′ (forward) and 5′CTGTTGATCTTGATGGCGGC–3′ (reverse); and HK3: 5′-CCTCACTGCGTGTTTTGTGG-3′ (forward) and 5′-GGGCAGCAAAGTCAAAGAGC-3′ (reverse); and CPT1B: 5′-TGGGTTCCTCCTTCTGCAAC-3′ (forward) and 5′-CGGAAGAAGAAGATGCCCGT-3′ (reverse); and CYP26A1: 5′-TGCATCCAGGTTCAGCTTCA-3′ (forward) and 5′-CAGGATACACGGTGGGACTG-3′ (reverse); and HAO2: 5′-CTGAGGTGGACACCAGAACC-3′ (forward) and 5′-TGCTGTGCTCATTTCCCCAT-3′ (reverse); GAPDH: 5′-AAAAGCATCACCCGGAGGAGAA-3′ (forward) and 5′-AAGGAAATGAATGGGCAGCCG-3′ (reverse).

### Statistical analysis

Chi-square test was conducted to detect the difference between two groups of patients with regard to clinicopathological features; differences in risk score between various cllinicopathological parameters were tested by Student’s t test. All statistical analysis were performed using GraphPad Prism7.0, SPSS version 16.0 software (SPSS Inc., Chicago, IL, USA); all data for bioinformatic analysis in the study were processed by Software R (version 3.6.1, https://www.r-project.org/). Data are presented as the mean ± SEM. If not specified above, *P* < 0.05 was regarded as statistically significant.

## Supplementary information

Supplementary Figure 1. Kaplan-Meier curves for prognostic value of ten genes constructing the model. (A) ALDH6A1. (B) FBP1. (C) HAO2. (D) PSAT1. (E) TYMP. (F) CPT1B. (G) HK3. (H) IL4I1. (I) P4HA3. (J) CYP26A1. Data was retrieved from the ONCOLNC database.

Supplementary Figure 2. Time-dependent ROC analysis of the risk score model in ccRCC. (A) 1-year performance as the risk score stratified by quartiles. (B) 3-year performance as the risk score stratified by quartiles. (C) 5-year performance as the risk score stratified by quartiles. (D) 1-year performance of risk score based on CPTAC validation set. (E) 3-year performance of risk score based on CPTAC validation set.

Supplementary Figure 3. A nomogram predicting overall survival for ccRCC patients. Every parameters matches a ruler, thus corresponding to a point. Overall survival was predicted for each patient based on total points.

Supplementary Figure 4. The expression of the ten predictive genes in cancer. (A) The expression profiles of the ten genes in the Oncomine database (http://www.oncomine.org/resource/main.html). Data of IL4I1, CPT1B, and CYP26A1 were not found in the database. (B) The expression profiles of the ten genes in the TIMER database (http://cistrome.shinyapps.io/timer/). Data of CPT1B was not found in the database.

Supplementary Figure 5. The functional annotation of this ten-gene signature. (A) Top 20 functional annotation of downregulated genes in high risk patients. (B) Top 20 functional annotation of upregulated genes in high risk patients.

Supplementary Figure 6. Flowchart detailing the overall study design at each stage.

Supplementary Table 1

Supplementary Table 2

## Data Availability

The datasets generated during and/or analysed during the current study are available from the corresponding author on reasonable request.
