# Supplementary figures and images for: A cluster of metabolism-related genes predict prognosis and progression of clear cell renal cell carcinoma
Source: Sci Rep. 2020 Jul 31;10:12949. doi: 10.1038/s41598-020-67760-6 (PMC7395775; doi:10.1038/s41598-020-67760-6)

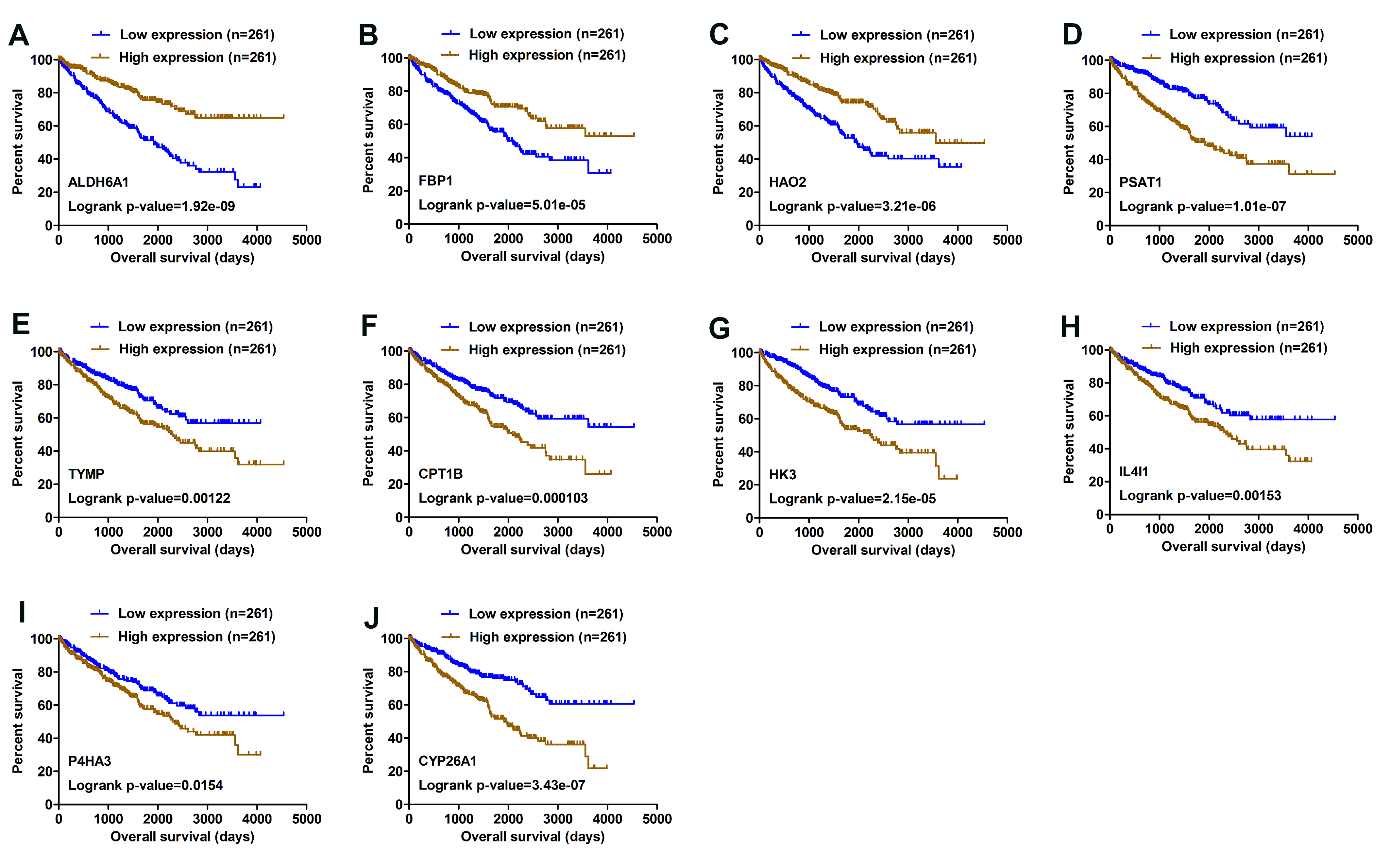

Supplement: Supplementary file 1 — Supplementary Figure 1. Kaplan-Meier curves for prognostic value of ten genes constructing the model. (A) ALDH6A1. (B) FBP1. (C) HAO2. (D) PSAT1. (E) TYMP. (F) CPT1B. (G) HK3. (H) IL4I1. (I) P4HA3. (J) CYP26A1. Data was retrieved from the ONCOLNC database. [file 41598_2020_67760_MOESM1_ESM.tif]

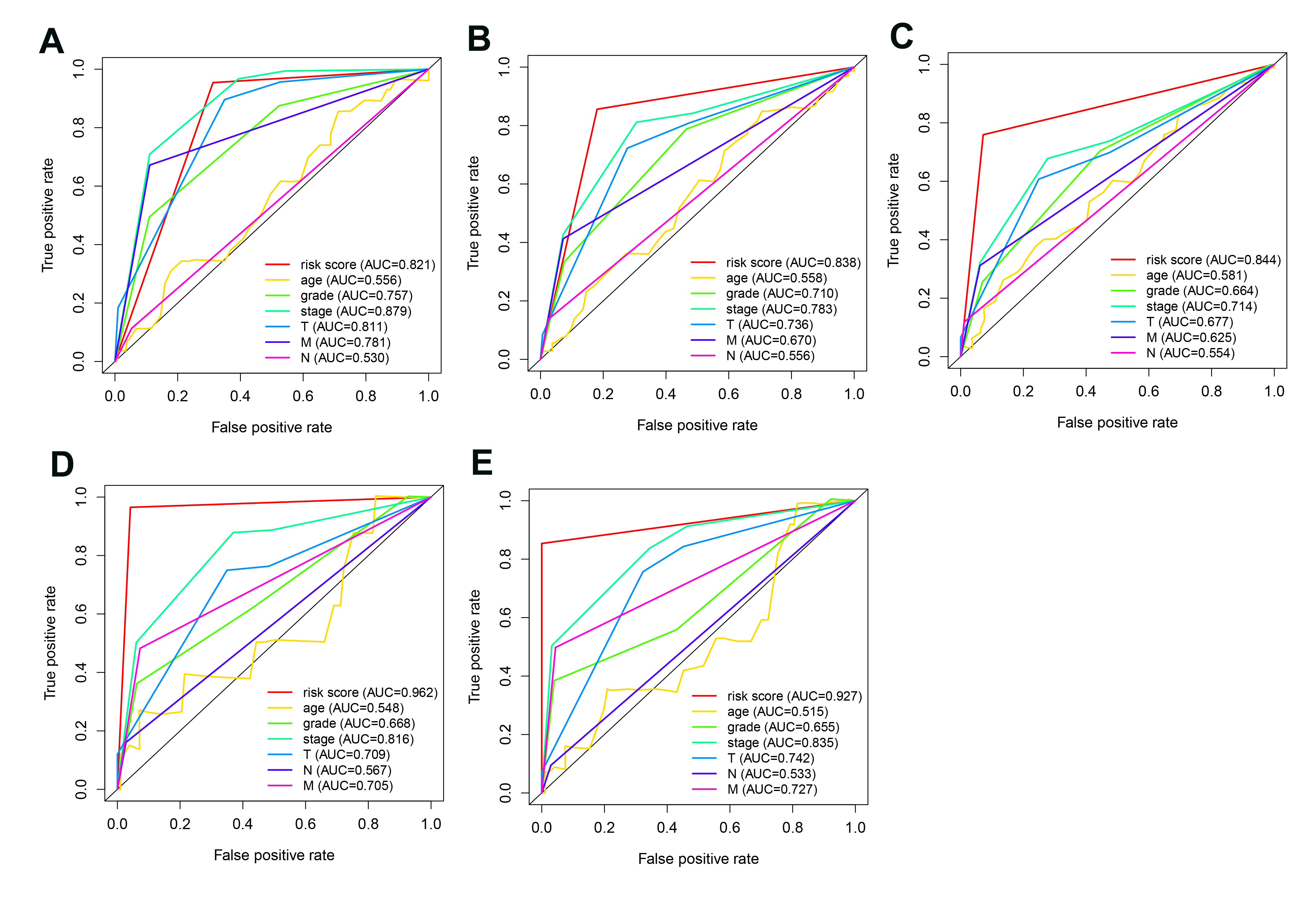

Supplement: Supplementary file 2 — Supplementary Figure 2. Time-dependent ROC analysis of the risk score model in ccRCC. (A) 1-year performance as the risk score stratified by quartiles. (B) 3-year performance as the risk score stratified by quartiles. (C) 5-year performance as the risk score stratified by quartiles. (D) 1-year performance of risk score based on CPTAC validation set. (E) 3-year performance of risk score based on CPTAC validation set. [file 41598_2020_67760_MOESM2_ESM.tif]

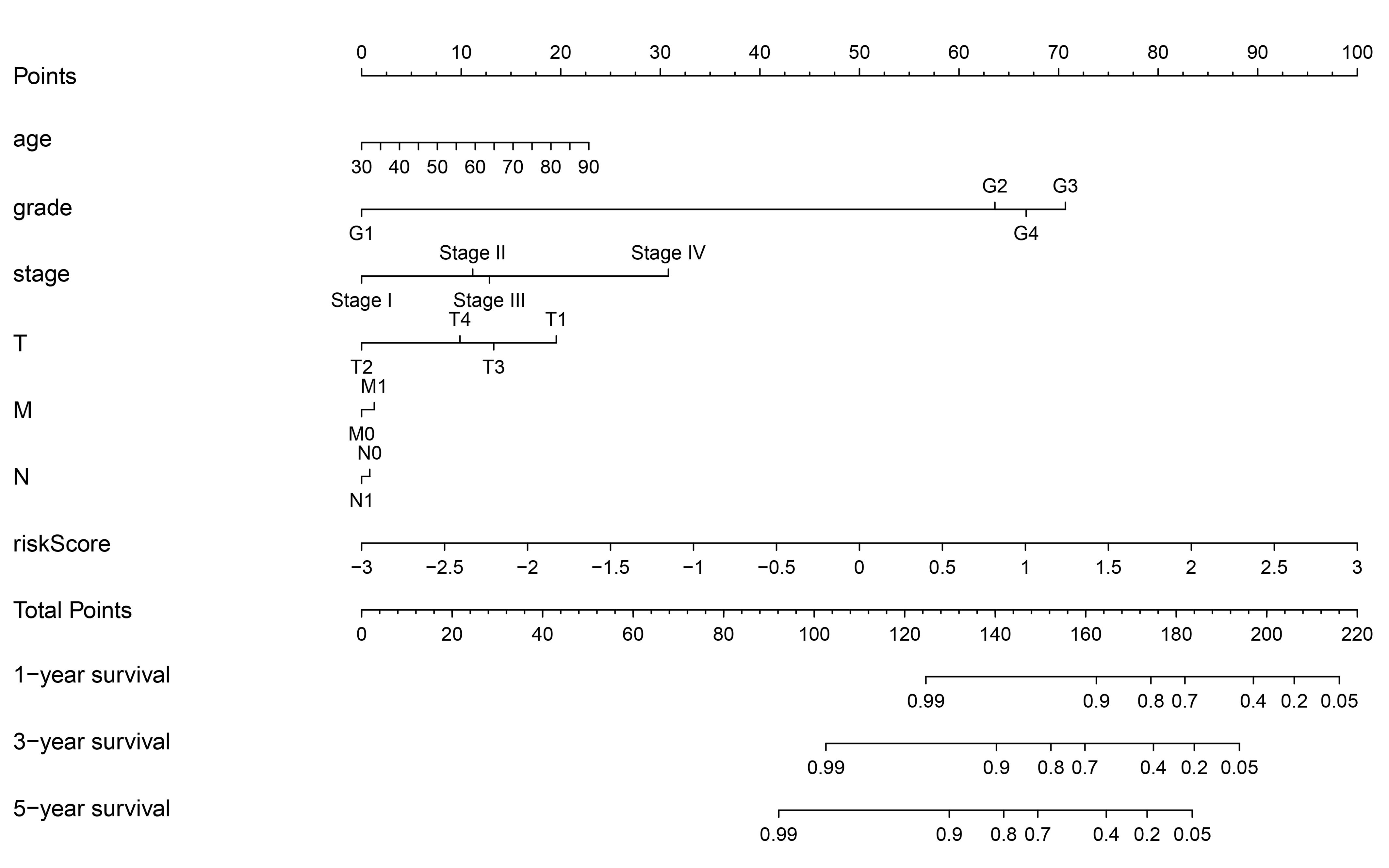

Supplement: Supplementary file 3 — Supplementary Figure 3. A nomogram predicting overall survival for ccRCC patients. Every parameters matches a ruler, thus corresponding to a point. Overall survival was predicted for each patient based on total points. [file 41598_2020_67760_MOESM3_ESM.tif]

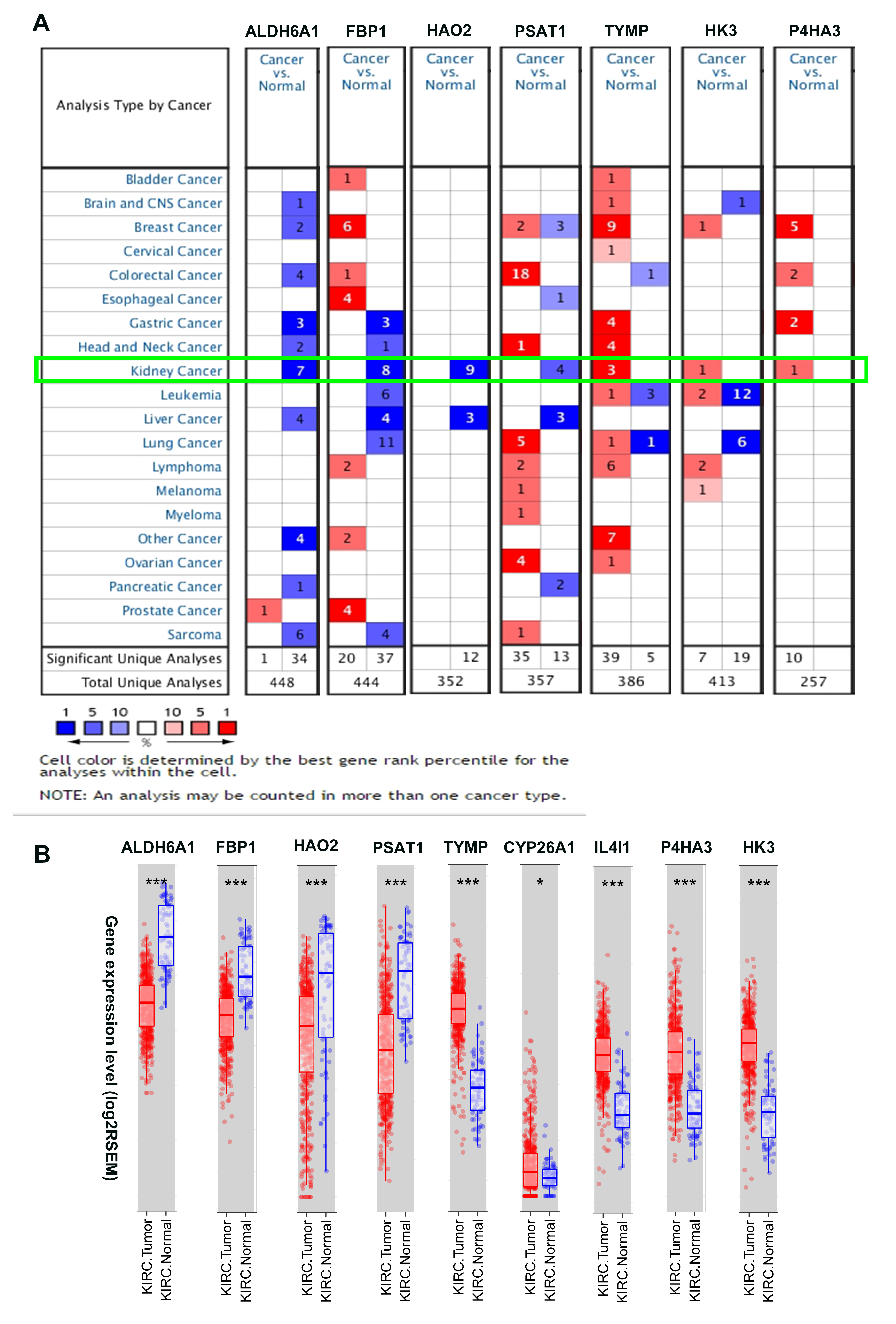

Supplement: Supplementary file 4 — Supplementary Figure 4. The expression of the ten predictive genes in cancer. (A) The expression profiles of the ten genes in the Oncomine database (http://www.oncomine.org/resource/main.html). Data of IL4I1, CPT1B, and CYP26A1 were not found in the database. (B) The expression profiles of the ten genes in the TIMER database (http://cistrome.shinyapps.io/timer/). Data of CPT1B was not found in the database. [file 41598_2020_67760_MOESM4_ESM.tif]

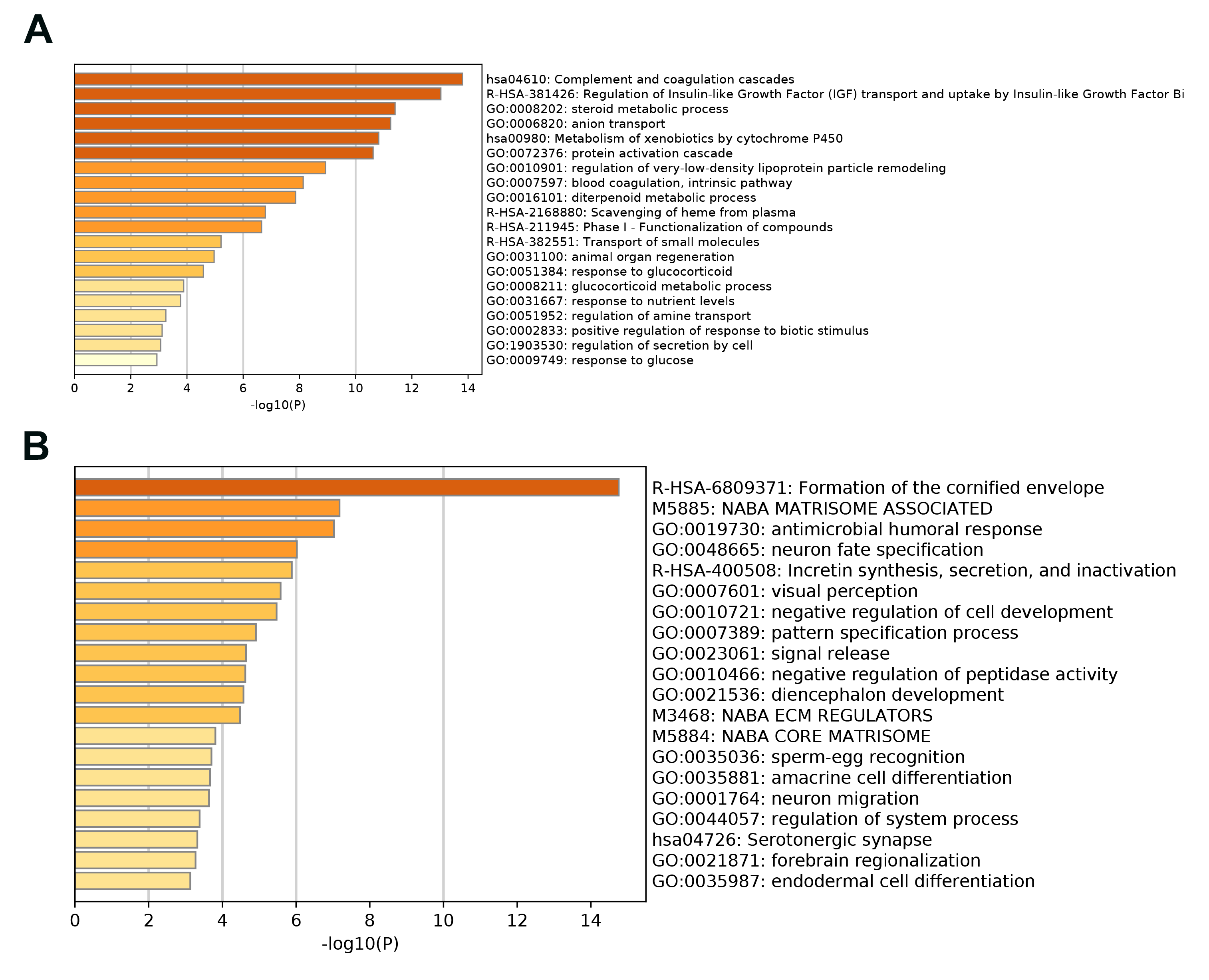

Supplement: Supplementary file 5 — Supplementary Figure 5. The functional annotation of this ten-gene signature. (A) Top 20 functional annotation of downregulated genes in high risk patients. (B) Top 20 functional annotation of upregulated genes in high risk patients. [file 41598_2020_67760_MOESM5_ESM.tif]

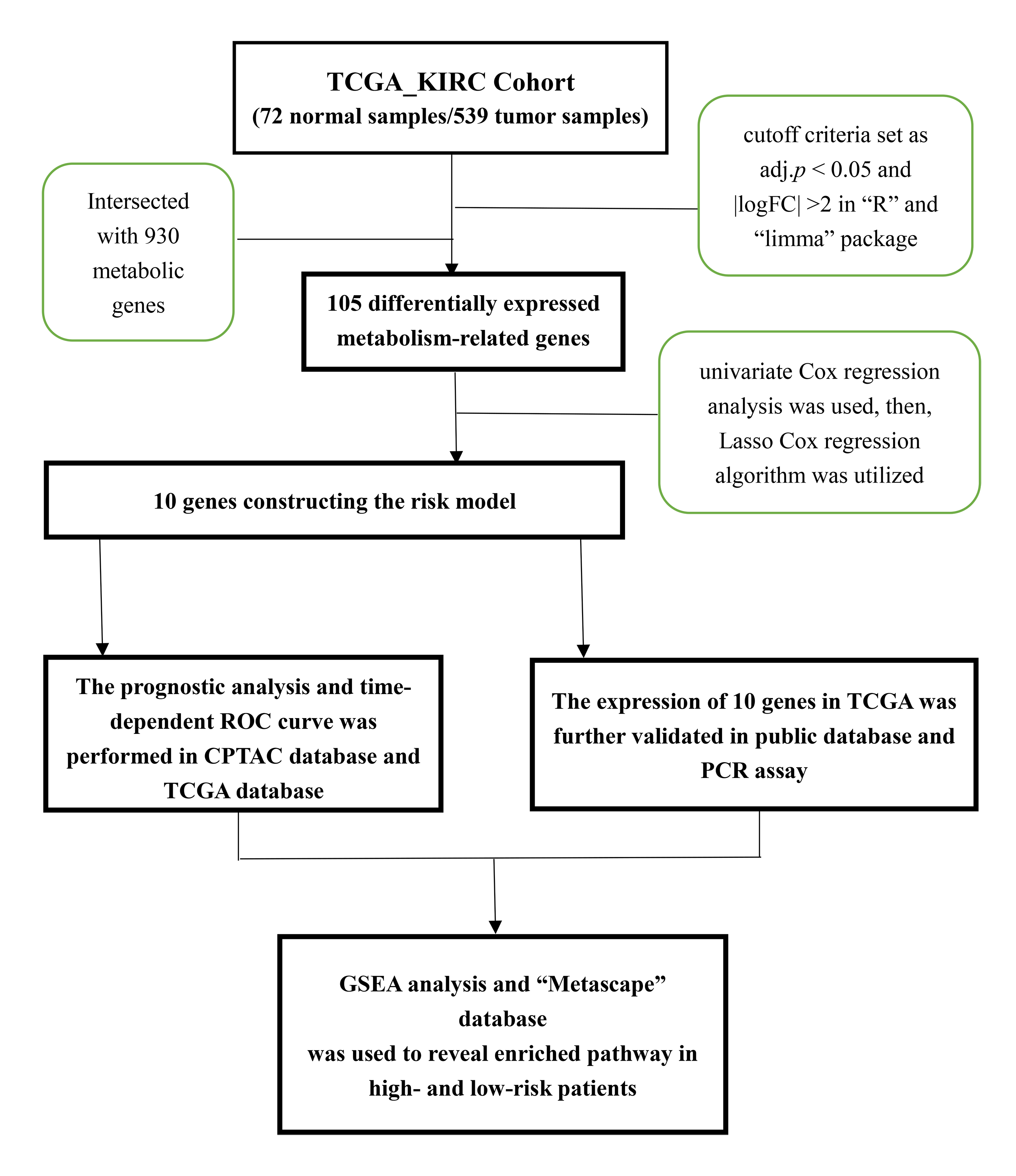

Supplement: Supplementary file 6 — Supplementary Figure 6. Flowchart detailing the overall study design at each stage. [file 41598_2020_67760_MOESM6_ESM.tif]
