# Supplementary material for: A cluster of metabolism-related genes predict prognosis and progression of clear cell renal cell carcinoma
Source: Sci Rep. 2020 Jul 31;10:12949. doi: 10.1038/s41598-020-67760-6 (PMC7395775; doi:10.1038/s41598-020-67760-6)
Supplement: Supplementary file 7 — Supplementary Table 1 [file 41598_2020_67760_MOESM7_ESM.doc]

**Supplementary Table 1. Differentially expressed metabolism-related genes between normal samples and tumor samples in TCGA_KIRC cohort.**

| **Gene** | **Log FC** | **P Value** | **FDR** | **Gene** | **Log FC** | **P Value** | **FDR** |
| --- | --- | --- | --- | --- | --- | --- | --- |
| ACPP | -5.493050301 | 3.10E-42 | 3.73E-40 | CYP4F20 | -4.997459501 |  | 3.61E-17 |
| TYRP1 | -5.351024065 | 1.82E-51 | 3.07E-49 | ACOT12 | -4.808514851 | 4.32E-72 | 1.82E-69 |
| GGT6 | -5.082951965 | 3.21E-55 | 6.76E-53 | ALDOB | -4.775378811 | 2.09E-20 | 6.28E-20 |
| HPD | -4.975907976 | 7.74E-26 | 3.49E-25 | CYP2B6 | -4.732782598 | 1.96E-41 | 2.06E-39 |
| ENPP6 | -4.942958935 | 4.54E-35 | 6.38E-34 | UGT2A1 | -4.609901078 | 5.75E-32 | 4.44E-31 |
| NOS1 | -4.348140151 | 1.54E-29 | 9.12E-29 | PIK3C2G | -4.005045239 | 1.33E-91 | 1.12E-88 |
| UPP2 | -3.805822914 | 7.74E-29 | 4.32E-28 | SULT2B1 | -3.260641036 | 6.37E-13 | 1.25E-12 |
| ADH1C | -3.527081921 | 8.42E-41 | 7.10E-39 | CYP1A1 | -3.092958235 | 4.58E-28 | 2.33E-27 |
| CA8 | -3.413443776 | 2.44E-38 | 9.62E-37 | ALDH6A1 | -2.850773592 | 7.95E-34 | 8.60E-33 |
| G6PC | -3.31855592 | 4.61E-13 | 9.07E-13 | DNMT3L | -2.765728254 | 8.98E-18 | 2.30E-17 |
| GSTM3 | -3.307325411 | 4.21E-41 | 3.94E-39 | HMGCS2 | -2.654943571 | 1.79E-31 | 1.27E-30 |
| DAO | -3.061685113 | 1.16E-11 | 2.18E-11 | ENTPD3 | -2.513325434 | 9.12E-38 | 2.85E-36 |
| DEGS2 | -3.04852934 | 9.17E-30 | 5.48E-29 | CYP4F3 | -2.290780028 | 2.07E-11 | 3.87E-11 |
| PAH | -2.984986581 | 6.79E-15 | 1.47E-14 | AGMAT | -2.274517559 | 5.88E-08 | 9.16E-08 |
| CEL | -2.923965793 | 5.21E-35 | 7.20E-34 | PLA2G3 | -2.145377087 | 4.10E-43 | 5.76E-41 |
| RDH8 | -2.901039864 | 8.54E-27 | 4.09E-26 | INPP5J | -2.13341984 | 7.12E-64 | 2.00E-61 |
| PDE1A | -2.876696455 | 2.89E-39 | 1.43E-37 | OGDHL | -2.02369066 | 1.50E-30 | 9.75E-30 |
| PIPOX | -2.816482215 | 8.50E-10 | 1.46E-09 | UGT2B11 | 2.013479485 | 2.13E-05 | 2.87E-05 |
| CKM | -2.801217232 | 1.68E-16 | 4.02E-16 | CHST13 | 2.016681362 | 5.29E-22 | 1.75E-21 |
| ABAT | -2.795511841 | 2.05E-25 | 8.83E-25 | SULT1A3 | 2.058658146 | 7.53E-15 | 1.63E-14 |
| PCK1 | -2.707935564 | 3.76E-19 | 1.07E-18 | CYP2C8 | 2.072198393 | 4.98E-11 | 9.00E-11 |
| ACSL6 | -2.655486828 | 4.60E-37 | 1.10E-35 | SORD | -2.023607052 | 1.93E-24 | 7.50E-24 |
| ADH1B | -2.633973947 | 7.11E-34 | 7.78E-33 | TYMS | 2.007272759 | 1.43E-36 | 2.87E-35 |
| ASS1 | -2.606623742 | 2.32E-15 | 5.20E-15 | CD38 | 2.036326079 | 7.46E-18 | 1.93E-17 |
| PNMT | -2.595093942 | 8.69E-32 | 6.37E-31 | IL4I1 | 2.100830401 | 2.42E-27 | 1.18E-26 |
| PLCL1 | -2.557987829 | 7.59E-38 | 2.46E-36 | ASMT | 2.100875234 | 2.93E-16 | 6.91E-16 |
| PSAT1 | -2.516891591 | 4.15E-24 | 1.58E-23 | TYMP | 2.180600394 | 4.69E-37 | 1.10E-35 |
| ADH6 | -2.467180333 | 1.09E-09 | 1.86E-09 | ALDOC | 2.184983742 | 4.42E-25 | 1.81E-24 |
| GPAT3 | -2.438795867 | 3.30E-35 | 4.80E-34 | PFKFB4 | 2.197805526 | 2.61E-34 | 3.10E-33 |
| GMPR | -2.410535174 | 3.39E-35 | 4.84E-34 | HMOX1 | 2.295491785 | 1.97E-32 | 1.63E-31 |
| LDHD | -2.38130551 | 1.43E-31 | 1.02E-30 | ADSSL1 | 2.318269056 | 4.48E-21 | 1.41E-20 |
| HAO2 | -2.318381708 | 3.14E-10 | 5.49E-10 | CPT1B | 2.32025423 | 2.04E-10 | 3.60E-10 |
| GLDC | -2.313654412 | 2.37E-36 | 4.44E-35 | HK3 | 2.342038899 | 6.24E-34 | 6.92E-33 |
| FMO5 | -2.279664278 | 1.80E-37 | 5.05E-36 | CYP3A5 | 2.352846426 | 9.57E-22 | 3.12E-21 |
| FBP1 | -2.275883753 | 4.77E-26 | 2.21E-25 | SDS | 2.389878704 | 1.86E-39 | 1.05E-37 |
| MIOX | -2.194380211 | 2.88E-06 | 4.16E-06 | RPE65 | 2.405690825 | 3.59E-20 | 1.06E-19 |
| TH | -2.161042367 | 1.02E-24 | 4.12E-24 | LPCAT1 | 2.415050303 | 3.13E-36 | 5.50E-35 |
| PCK2 | -2.157585 | 1.11E-09 | 1.89E-09 | GAD2 | 2.458120634 | 2.20E-12 | 4.23E-12 |
| TREH | -2.125637184 | 2.55E-18 | 6.85E-18 | CHIT1 | 2.479417958 | 1.23E-21 | 3.93E-21 |
| AGXT | -2.101664505 | 1.16E-12 | 2.25E-12 | ENPP3 | 5.473267771 | 8.56E-34 | 9.13E-33 |
| PLA2G5 | 2.619361742 | 4.29E-26 | 2.00E-25 | DHRS9 | 2.62839331 | 1.93E-20 | 5.80E-20 |
| CYP2F1 | 2.678308125 | 3.16E-05 | 4.20E-05 | GAL3ST1 | 2.809308569 | 2.39E-30 | 1.51E-29 |
| IDO1 | 2.942387051 | 2.43E-36 | 4.46E-35 | P4HA3 | 3.118553655 | 6.50E-29 | 3.68E-28 |
| ITPKA | 3.200056064 | 2.01E-27 | 9.87E-27 | ADCY2 | 3.207963429 | 1.44E-06 | 2.11E-06 |
| UGT2B17 | 3.251285434 | 1.33E-09 | 2.26E-09 | NNMT | 3.408868309 | 9.25E-36 | 1.50E-34 |
| CYP2A6 | 3.432815345 | 6.48E-07 | 9.63E-07 | CP | 3.460042046 | 1.15E-24 | 4.60E-24 |
| PLA2G2D | 3.614159809 | 2.39E-19 | 6.84E-19 | ADCY8 | 3.838816563 | 6.60E-05 | 8.66E-05 |
| ALOX15B | 3.882118415 | 2.52E-34 | 3.03E-33 | CYP2J2 | 3.998366204 | 1.17E-30 | 7.69E-30 |
| BAAT | 4.059725676 | 2.55E-11 | 4.73E-11 | PLA2G7 | 4.146814033 | 1.43E-36 | 2.87E-35 |
| CA9 | 4.702063723 | 5.35E-38 | 1.88E-36 | HK2 | 4.879806935 | 2.37E-37 | 6.45E-36 |
| CYP26A1 | 2.276066142 | 0.01147881 | 0.0133287 | PRODH2 | -2.03662932 | 0.000158108 | 0.000203179 |
| UGT1A10 | 2.195208403 | 0.001147985 | 0.001427362 |  |  |  |  |
