# Supplementary material for: A cluster of metabolism-related genes predict prognosis and progression of clear cell renal cell carcinoma
Source: Sci Rep. 2020 Jul 31;10:12949. doi: 10.1038/s41598-020-67760-6 (PMC7395775; doi:10.1038/s41598-020-67760-6)
Supplement: Supplementary file 8 — Supplementary Table 2 [file 41598_2020_67760_MOESM8_ESM.doc]

**Supplementary Table 2. Clinical information of CPTAC_ccRCC Cohort.**

| **case_id** | **Futime**  **(days)** | **fustate** | **gender** | **age** | **grade** | **TNM stage** | **T stage** | **N stage** | **M stage** |
| --- | --- | --- | --- | --- | --- | --- | --- | --- | --- |
| C3L-00004 | 384.0 | 0 | Male | 72 | G3 | Stage III | pT3 | pNX | cM0 |
| C3L-00010 | 879.0 | 0 | Male | 30 | G3 | Stage I | pT1b | pN0 | cM0 |
| C3L-00011 | 229.0 | 1 | Female | 63 | G4 | Stage IV | pT3a | pNX | cM1 |
| C3L-00026 | 1066.0 | 0 | Female | 65 | G3 | Stage I | pT1a | pNX | cM0 |
| C3L-00079 | 245.0 | 1 | Male | 49 | G3 | Stage III | pT3a | pN1 | cM0 |
| C3L-00088 | 687.0 | 1 | Male | 72 | G2 | Stage III | pT3a | pNX | cM0 |
| C3L-00096 | 68.0 | 1 | Male | 52 | G4 | Stage IV | pT3a | pN0 | cM1 |
| C3L-00097 | 1081.0 | 0 | Male | 59 | G2 | Stage I | pT1a | pNX | cM0 |
| C3L-00103 | 1071.0 | 0 | Male | 56 | G3 | Stage III | pT3b | pN0 | cM0 |
| C3L-00183 | 227.0 | 0 | Female | 33 | G2 | Stage III | pT3a | pNX | cM0 |
| C3L-00359 | 1064.0 | 0 | Female | 73 | G1 | Stage I | pT1a | pNX | cM0 |
| C3L-00360 | 1014.0 | 0 | Male | 72 | G3 | Stage II | pT2a | pNX | cM0 |
| C3L-00369 | 91.0 | 1 | Male | 89 | G2 | Stage III | pT3a | pNX | cMx |
| C3L-00416 | 608.0 | 0 | Male | 62 | G4 | Stage III | pT3b | pNX | cMx |
| C3L-00418 | 1063.0 | 0 | Female | 56 | G2 | Stage III | pT3a | pNX | cM0 |
| C3L-00447 | 671.0 | 0 | Male | 35 | G3 | Stage I | pT1b | pNX | cM0 |
| C3L-00448 | 1072.0 | 0 | Male | 66 | G2 | Stage I | pT1a | pNX | cM0 |
| C3L-00561 | 1044.0 | 0 | Male | 50 | G4 | Stage III | pT3b | pN0 | cM0 |
| C3L-00581 | 704.0 | 0 | Male | 52 | G2 | Stage II | pT2b | pNX | cM0 |
| C3L-00583 | 301.0 | 0 | Male | 55 | G3 | Stage I | pT1a | pNX | cM0 |
| C3L-00606 | 672.0 | 0 | Female | 75 | G3 | Stage III | pT3a | pN0 | cM0 |
| C3L-00607 | 727.0 | 0 | Male | 66 | G3 | Stage III | pT3a | pNX | cMx |
| C3L-00610 | 723.0 | 0 | Female | 57 | G3 | Stage I | pT1b | pNX | cMx |
| C3L-00765 | 587.0 | 0 | Female | 61 | G2 | Stage I | pT1b | pNX | cMx |
| C3L-00766 | 266.0 | 0 | Female | 57 | G2 | Stage I | pT1a | pNX | cMx |
| C3L-00790 | 441.0 | 0 | Male | 44 | G2 | Stage I | pT1b | pNX | cMx |
| C3L-00791 | 707.0 | 0 | Male | 76 | G3 | Stage III | pT1b | pN1 | cMx |
| C3L-00792 | 700.0 | 0 | Female | >=90 | G3 | Stage II | pT2a | pNX | cMx |
| C3L-00796 | 333.0 | 0 | Male | 67 | G2 | Stage I | pT1b | pNX | cMx |
| C3L-00799 | 495.0 | 0 | Male | 41 | G2 | Stage I | pT1a | pNX | cMx |
| C3L-00800 | 343.0 | 0 | Male | 41 | G2 | Stage I | pT1a | pNX | cMx |
| C3L-00812 | 720 | 0 | Male | 74 | G2 | Stage I | pT1a | pNX | cMx |
| C3L-00813 | 360 | 0 | Male | 61 | G3 | Stage III | pT3a | pNX | cMx |
| C3L-00814 | 15.0 | 0 | Male | 83 | G2 | Stage I | pT1a | pNX | cMx |
| C3L-00817 | 670.0 | 0 | Male | 80 | G2 | Stage I | pT1a | pNX | cMx |
| C3L-00902 | 609.0 | 0 | Female | 69 | G1 | Stage I | pT1b | pNX | cM0 |
| C3L-00907 | 693.0 | 0 | Male | 55 | G2 | Stage I | pT1b | pNX | cMx |
| C3L-00908 | 648.0 | 0 | Female | 60 | G3 | Stage II | pT2b | pNX | cMx |
| C3L-00910 | 681.0 | 0 | Female | 75 | G3 | Stage I | pT1b | pNX | cM0 |
| C3L-00917 | 684.0 | 0 | Male | 37 | G3 | Stage I | pT1a | pNX | cM0 |
| C3L-01281 | 685.0 | 0 | Male | 66 | G4 | Stage IV | pT3a | pNX | cM1 |
| C3L-01283 | 673.0 | 0 | Male | 37 | G3 | Stage I | pT1a | pNX | cM0 |
| C3L-01286 | 698.0 | 0 | Male | 58 | G3 | Stage III | pT3a | pNX | cM0 |
| C3L-01287 | 630.0 | 1 | Male | 60 | G4 | Stage IV | pT3a | pNX | cM1 |
| C3L-01288 | 703.0 | 0 | Female | 70 | G1 | Stage I | pT1a | pNX | cM0 |
| C3L-01302 | 53.0 | 1 | Female | 71 | G4 | Stage III | pT3a | pN0 | cM0 |
| C3L-01313 | 674.0 | 0 | Male | 63 | G4 | Stage III | pT3a | pNX | cMx |
| C3L-01352 | 232.0 | 0 | Female | 76 | G2 | Stage I | pT1a | pNX | cMx |
| C3L-01553 | 384.0 | 0 | Male | 59 | G2 | Stage I | pT1a | pNX | cMx |
| C3L-01557 | 329.0 | 0 | Male | 51 | G3 | Stage III | pT3a | pN0 | cMx |
| C3L-01560 | 250.0 | 0 | Female | 54 | G3 | Stage I | pT1a | pNX | cMx |
| C3L-01603 | 690.0 | 0 | Male | 63 | G2 | Stage I | pT1a | pNX | cMx |
| C3L-01607 | 646.0 | 0 | Male | 83 | G3 | Stage III | pT3a | pNX | cMx |
| C3L-01836 | 426.0 | 0 | Male | 47 | G3 | Stage I | pT1b | pNX | cMx |
| C3L-01861 | 701.0 | 0 | Male | 57 | G3 | Stage I | pT1a | pNX | cMx |
| C3L-01882 | 350.0 | 0 | Male | 48 | G2 | Stage I | pT1a | pN0 | cM0 |
| C3L-01885 | 342.0 | 0 | Male | 67 | G2 | Stage I | pT1a | pNX | cM0 |
| C3N-00168 | 826.0 | 0 | Male | 47 | G3 | Stage I | pT1b | pN0 | cM0 |
| C3N-00177 | 841.0 | 0 | Male | 52 | G2 | Stage III | pT3 | pN0 | cM0 |
| C3N-00194 | 208.0 | 0 | Female | 51 | G3 | Stage IV | pT2b | pNX | cMx |
| C3N-00242 | 624.0 | 0 | Male | 69 | G2 | Stage I | pT1a | pNX | cMx |
| C3N-00244 | 728.0 | 0 | Male | 46 | G2 | Stage I | pT1a | pNX | cMx |
| C3N-00246 | 700.0 | 0 | Male | 44 | G2 | Stage I | pT1a | pNX | cMx |
| C3N-00305 | 765.0 | 0 | Female | 60 | G1 | Stage I | pT1a | pNX | cMx |
| C3N-00310 | 759.0 | 0 | Male | 84 | G2 | Stage III | pT3a | pNX | cMx |
| C3N-00313 | 1077.0 | 0 | Female | 31 | G2 | Stage I | pT1b | pNX | cMx |
| C3N-00314 | 62.0 | 1 | Male | 78 | G2 | Stage I | pT1b | pNX | cMx |
| C3N-00315 | 740.0 | 0 | Male | 68 | G3 | Stage I | pT1b | pNX | cMx |
| C3N-00317 | 763.0 | 0 | Female | 74 | G2 | Stage I | pT1b | pNX | cMx |
| C3N-00320 | 765.0 | 0 | Male | 67 | G2 | Stage III | pT3a | pNX | cMx |
| C3N-00380 | 662.0 | 0 | Female | 57 | G2 | Stage I | pT1b | pNX | cM0 |
| C3N-00390 | 687.0 | 0 | Male | 58 | G2 | Stage IV | pT3a | pNX | cM1 |
| C3N-00435 | 7.0 | 0 | Male | 72 | G3 | Stage III | pT3 | pN1 | cM0 |
| C3N-00437 | 725.0 | 0 | Female | 69 | G3 | Stage III | pT3a | pNX | cMx |
| C3N-00491 | 77.0 | 1 | Male | 54 | G2 | Stage IV | pT1b | pNX | cM1 |
| C3N-00492 | 708.0 | 0 | Female | 49 | G3 | Stage II | pT2b | pNX | cMx |
| C3N-00494 | 702.0 | 0 | Male | 66 | G2 | Stage I | pT1b | pNX | cMx |
| C3N-00495 | 695.0 | 0 | Male | 67 | G2 | Stage I | pT1a | pNX | cMx |
| C3N-00573 | 6.0 | 0 | Male | 61 | G2 | Stage II | pT2b | pN0 | cM0 |
| C3N-00577 | 397.0 | 0 | Male | 72 | G3 | Stage IV | pT3 | pN1 | cM0 |
| C3N-00733 | 629.0 | 0 | Male | 56 | G2 | Stage III | pT3a | pNX | cMx |
| C3N-00831 | 374.0 | 1 | Male | 52 | G2 | Stage II | pT2a | pNX | cM1 |
| C3N-00832 | 784.0 | 0 | Male | 79 | G2 | Stage I | pT1b | pNX | cMx |
| C3N-00310 | 759.0 | 0 | Male | 84 | G2 | Stage III | pT3a | pNX | cMx |
| C3N-00834 | 770.0 | 0 | Male | 65 | G1 | Stage I | pT1b | pNX | cMx |
| C3N-00852 | 619.0 | 0 | Male | 62 | G3 | Stage III | pT3a | pNX | cM0 |
| C3N-00953 | 684.0 | 0 | Male | 41 | G3 | Stage II | pT2a | pNX | cMx |
| C3N-01175 | 729.0 | 0 | Female | 60 | G2 | Stage III | pT3a | pNX | cMx |
| C3N-01176 | 770.0 | 0 | Male | 71 | G2 | Stage III | pT3a | pNX | cM1 |
| C3N-01178 | 763.0 | 0 | Female | 58 | G2 | Stage III | pT3a | pNX | cMx |
| C3N-01179 | 754.0 | 0 | Male | 72 | G2 | Stage III | pT3a | pNX | cMx |
| C3N-01180 | 746.0 | 0 | Male | 51 | G2 | Stage I | pT1b | pNX | cMx |
| C3N-01200 | 47.0 | 1 | Female | 56 | G3 | Stage IV | pT4 | pN0 | cM1 |
| C3N-01213 | 653.0 | 0 | Male | 68 | G1 | Stage III | pT3a | pNX | cM0 |
| C3N-01214 | 652.0 | 0 | Male | 60 | G2 | Stage II | pT2a | pN0 | cM0 |
| C3N-01220 | 632.0 | 1 | Male | 51 | G4 | Stage IV | pT3a | pNX | cM1 |
| C3N-01261 | 680.0 | 0 | Male | 67 | G1 | Stage I | pT1b | pNX | cM0 |
| C3N-01361 | 784.0 | 0 | Male | 48 | G2 | Stage I | pT1a | pNX | cMx |
| C3N-01522 | 692.0 | 0 | Male | 80 | G2 | Stage I | pT1b | pN0 | cM0 |
| C3N-01524 | 694.0 | 0 | Male | 61 | G3 | Stage II | pT2a | pNX | cM0 |
| C3N-01646 | 742.0 | 0 | Male | 69 | G3 | Stage III | pT3a | pNX | cMx |
| C3N-01648 | 668.0 | 0 | Male | 69 | G2 | Stage II | pT2a | pNX | cMx |
| C3N-01649 | 733.0 | 0 | Male | 51 | G2 | Stage III | pT3a | pNX | cM1 |
| C3N-01651 | 732.0 | 0 | Male | 58 | G3 | Stage II | pT2a | pNX | cMx |
| C3N-01808 | 778.0 | 0 | Male | 47 | G2 | Stage I | pT1b | pNX | cMx |
